# Comparing the Effects of Isoflurane and Alpha Chloralose upon Mouse Physiology

**DOI:** 10.1371/journal.pone.0154936

**Published:** 2016-05-05

**Authors:** Lucie A. Low, Lucy C. Bauer, Brenda A. Klaunberg

**Affiliations:** 1 National Center for Complementary and Integrative Health, National Institutes of Health, Bethesda, Maryland, United States; 2 NIH Mouse Imaging Facility, National Institute of Neurological Disorders and Stroke, National Institutes of Health, Bethesda, Maryland, United States; Harvard University Faculty of Arts and Sciences, UNITED STATES

## Abstract

Functional magnetic resonance imaging of mice requires that the physiology of the mouse (body temperature, respiration and heart rates, blood pH level) be maintained in order to prevent changes affecting the outcomes of functional scanning, namely blood oxygenation level dependent (BOLD) measures and cerebral blood flow (CBF). The anesthetic used to sedate mice for scanning can have major effects on physiology. While alpha chloralose has been commonly used for functional imaging of rats, its effects on physiology are not well characterized in the literature for any species. In this study, we anesthetized or sedated mice with isoflurane or alpha chloralose for up to two hours, and monitored physiological parameters and arterial blood gasses. We found that, when normal body temperature is maintained, breathing rates for both drugs decrease over the course of two hours. In addition, alpha chloralose causes a substantial drop in heart rate and blood pH with severe hypercapnia (elevated blood CO_2_) that is not seen in isoflurane-treated animals. We suggest that alpha chloralose does not maintain normal mouse physiology adequately for functional brain imaging outcome measures.

## 1. Introduction

Functional magnetic resonance imaging (fMRI) monitors blood flow to the brain as a surrogate measure of regional brain activation. In humans, physiological changes that can influence blood flow, such as heart rate, breathing rate, body temperature and blood oxygen saturation levels, can be continuously and non-invasively measured. However, when scanning rodents for basic pre-clinical research, these measures can be challenging to monitor, and less amenable to correction. In particular, blood oxygenation and carbon dioxide levels are particularly important to monitor, as these can directly affect cerebral blood flow (CBF), and hence the outcome measures used in functional scanning [1–4].

In the rat, there have been a number of successful studies of rat brain activation using fMRI in both anesthetized [5–7] and awake [8–11] rats. For accurate fMRI results it is imperative to maintain normal physiology to minimize any potential effects on regional changes in CBF and blood oxygen level dependent (BOLD) contrast. A decrease in blood gas partial pressures of oxygen (hypoxemia) [12] or an increase in blood gas partial pressures of carbon dioxide (hypercapnia) will both increase cerebral blood flow [13]. Blood gas levels can be indirectly monitored by end tidal carbon dioxide measurement (capnography) in the rat, or by insertion of an arterial catheter for periodic blood gas sampling in anesthetized animals. Variations in acid-base status can have severe consequences for functional scanning because sustained deviations in pH can cause a cascade of metabolic effects, including changes in oxygen binding capacities [14]. Systemically, wide divergence of pH can have severe to life-threatening consequences including lethargy and coma (if blood becomes too acidic), or muscle twitching, tetany, and seizures (if blood becomes too basic).

The advent of genetic knockout technologies in mice has led to a window of opportunity in rodent scanning, where important information can be gleaned about brain processing in genetically modified mice. Functional MR scanning of these mice, combined with genetic and behavioral approaches, could powerfully combine to provide better translational animal models for basic and preclinical research. However, the physiological monitoring and homeostatic maintenance of mice under anesthesia is technically challenging. End tidal CO_2_ of the mouse cannot generally be measured accurately with standard available equipment, as the lung capacity of the mouse is too small (1.2–1.8 ml)[15]. Arterial catheters are invasive and challenging to implant in the mouse, and the volume of blood that can be taken safely is extremely limited. While volumes up to 150μl of blood can be taken from a 200g rat every 24 hours [16] (and NIH ARAC guidelines, http://oacu.od.nih.gov/ARAC/documents/Rodent_Bleeding.pdf), the entire circulating blood volume of a 20g mouse may only be 1.4 ml, and therefore taking even a small sample can result in significant changes in blood pressure and, hence, functional outcome measures [17–19].

To minimize stress in the noisy scanner environment and reduce movement artifact, rodents are often anesthetized. A variety of anesthetics and sedatives have been used for in-vivo imaging, including inhalation anesthetics such as isoflurane [20–22], sedatives such as medetomidine (Domitor^®^) or alpha chloralose [23–26] often combined with a paralytic such as pancuronium bromide [27, 28], and dissociative agents such as ketamine in combination with muscle relaxants such as xylazine [29, 30]. For functional scanning, alpha chloralose has been recommended as an ideal sedative for rodent studies as it preserves cortical blood flow signals and a good signal-to-noise ratio (SNR)[31]. However, alpha chloralose is associated with prolonged and poor recovery [32], so it is not generally allowed for longitudinal studies. Inhalant anesthetics such as isoflurane can depress cortical activity and can be potent vasodilators, potentially confounding blood flow signals [33–35]. Additionally, dissociative anesthetics such as ketamine may alter the function of neural signaling, inherently altering the desired outcome measure for functional imaging [36]. The use of anesthesia introduces many potential confounds into brain imaging in the rodent, so it is important for researchers to be aware of the effects of anesthesia regimen upon their experimental animal.

In this study, we investigated the physiological effects of isoflurane anesthesia and alpha chloralose sedation in the mouse up to 120 minutes post-induction, a time course similar to that used for functional scanning of the mouse brain. Breathing rate, heart rate and body temperature were monitored. Arterial blood pH, arterial blood oxygen and carbon dioxide partial pressures (P_a_O_2_ and P_a_CO_2_) were measured during, and/or at the end of anesthesia. We found that alpha chloralose causes considerable blood acidosis and cardiopulmonary depression beginning within 30 minutes after induction, and that isoflurane maintains better homeostasis. This information can help investigators choose appropriate anesthesia/sedation regimens for functional scanning.

## 2. Materials and Methods

### 2.1 Animals

C57Bl/6 male mice (Jackson), 28-46g, were used. All mice were housed on a 12-hour light/dark cycle in AAALAC-accredited NIH animal housing facilities with *ad libitum* access to food and water. Studies were approved by the National Institutes of Health National Institute of Neurological Disorders and Stroke (NINDS) Animal Care and Use Committee (ACUC) under protocol #1368–14. All efforts were taken to ameliorate animal suffering.

Anesthesia was induced by isoflurane and sedation by alpha chloralose. Animals were arbitrarily chosen to receive isoflurane or alpha chloralose (AC) on experimental days, which were counterbalanced between groups. Animals undergoing isoflurane anesthesia were not subsequently used for the alpha chloralose portion of the experiment. Euthanasia after alpha chloralose administration was performed by cervical dislocation.

### 2.2 Anesthetics

Mice were anesthetized with either isoflurane (n = 24–36, depending on outcome measure) or sedated with alpha chloralose (n = 15–17, depending on outcome measure). Isoflurane mice were induced in a chamber at 4–5% and maintained at 1.5–2% in 60% oxygen-supplemented air via nosecone [37]. Alpha chloralose mice were first induced with 4–5% isoflurane, and then given an 114mg/kg i.p. injection of alpha chloralose [38]. Mice were maintained under decreasing doses of isoflurane for approximately 15 minutes until the alpha chloralose took effect. 60% oxygen-supplemented air was provided via nosecone. Alpha chloralose was dissolved in an 80:20 mixture of 1XPBS (phosphate buffered saline):PEG (polyethylene glycol, Sigma). At 60 minutes, alpha chloralose-sedated mice were given an additional half-dose bolus. All animals were given 1ml of 0.9% saline i.p. or s.c. before physiological monitoring equipment was attached to maintain blood pressure, mitigate anticipated respiratory losses, and to aid tail arterial blood sampling.

### 2.3 Physiological monitoring

Physiological measures were taken every five minutes while the animal was under anesthesia. Breathing rate (breaths/min) was measured with a pneumatic pillow (SA Instruments, Inc., Stony Brook, NY) placed beneath the anesthetized animal. Core body temperature was measured by rectal probe and maintained between 36–37°C (±0.5°) with a water-heated temperature feedback system consisting of a polystat PPO Heated Bath and Digi-Sense temperature controller R/S (Cole-Parmer, Vernon Hills, Illinois). Heart rate was measured using mouse-specific echocardiogram (ECG) leads (SA Instruments, Inc. Stony Brook, NY). Leads were subcutaneously placed in the two axillary regions and right hind limb with caution to avoid local arteries or nerves. We chose non-invasive monitoring techniques compatible with MRI imaging.

Arterial samples for blood gas analysis were taken from the tail at various time intervals up to 120 minutes during anesthesia. The NIH Guideline for survival bleeding of rodents was followed (http://oacu.od.nih.gov/ARAC/documents/Rodent_Bleeding.pdf). Before sampling, depth of anesthesia was checked by gently pinching the hind paw toes and monitoring the withdrawal reflex. The tail was warmed to increase perfusion, the artery was nicked with a 19-gauge needle or scalpel, and 50–75μl of blood was immediately collected into a 100μl heparinized capillary tube. Direct pressure and elevation was applied to the tail immediately after sampling to achieve rapid hemostasis. A drop of cyanoacrylate tissue adhesive was applied to prevent any further blood loss. Blood pH, partial pressure of oxygen (P_a_O_2_), and partial pressure of carbon dioxide (P_a_CO_2_) were measured immediately using an ABL80 FLEX radiometer (Radiometer Medical ApS, Bronshoj, Denmark). Mice receiving isoflurane were sampled no more than twice (depending on weight) and mice receiving terminal alpha chloralose were sampled no more than 3 times during the 120 minute observation period, with any further samples taken at the terminal time point, or within 10 minutes prior.

All physiologic parameters (except pH and blood gas levels) were noted every 5 minutes for anesthesia durations up to 120 minutes. Mice were allowed to naturally recover from isoflurane anesthesia on a heated pad in home cages. Alpha chloralose is not approved for survival procedures; therefore mice were euthanized by cervical dislocation at the end of alpha chloralose recording periods.

### 2.4 Statistical analyses

2-way ANOVAs were performed using Prism 6 software, with treatment and time as factors. 2-tailed unpaired t-tests were performed to compare blood O_2_, CO_2_ and pH at the 120 minute time point.

## 3. Results

In general, alpha chloralose produced a lighter, less stable plane of sedation. Qualitative observation showed that AC-treated animals showed occasional startle responses to external stimuli such as loud noises, and variable withdrawal responses to toe pinch, used to check depth of anesthesia. These reflexes were absent in isoflurane-anesthetized animals.

### 3.1 Breathing rate under anesthesia or sedation decreases over time

**Fig 1:**
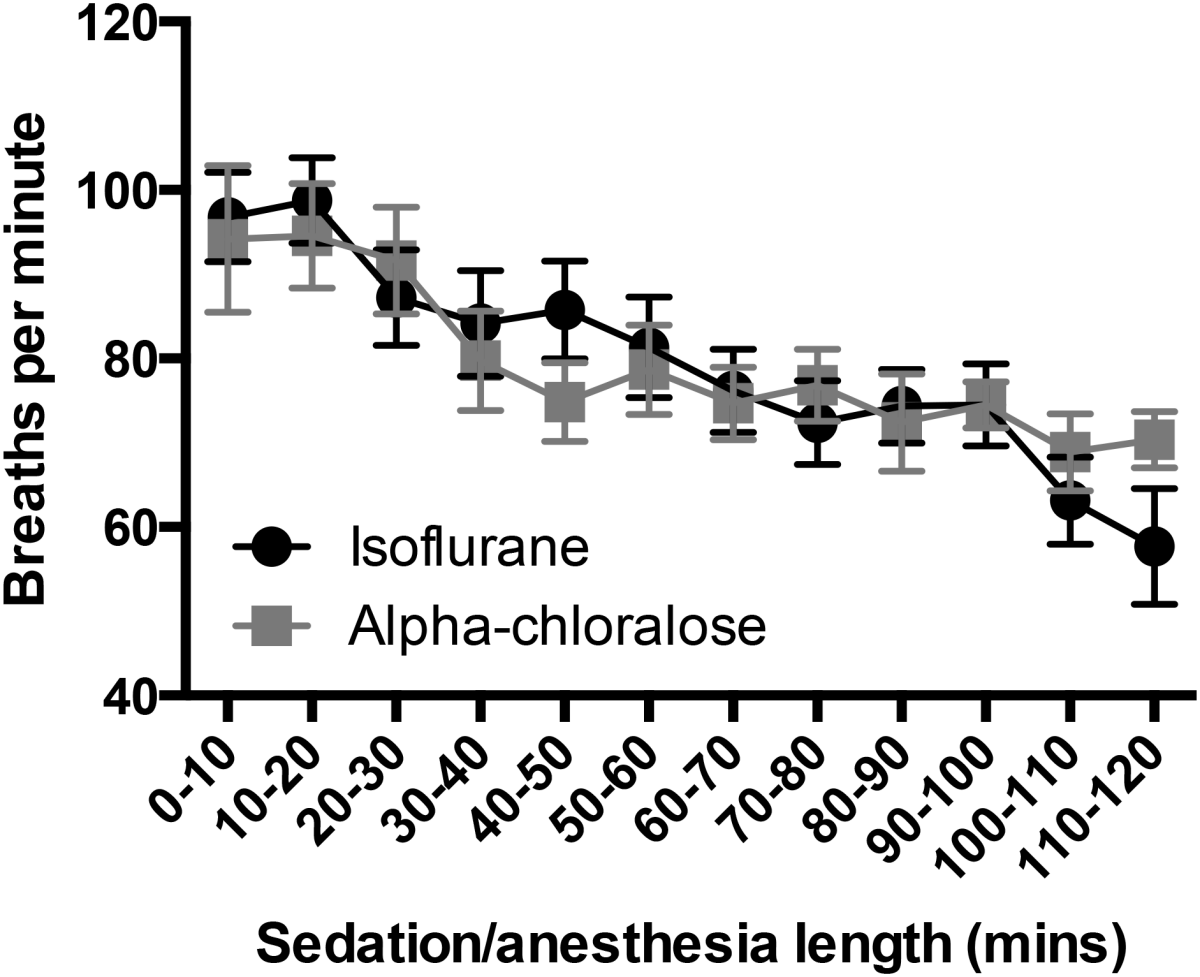
Breathing Rate decreases over time during prolonged anesthesia. There were no group differences between isoflurane and alpha chloralose-treated mice at any time (F_(1,386)_ = 0.001, p = 0.97), but there was a significant overall drop in breathing rates for both groups (F_(11,386)_ = 5.68, p<0.0001, n = 7–24 per time point).

Breathing rates fell over time under both isoflurane anesthesia and alpha chloralose sedation (F_(11,386)_ = 5.68, p<0.0001, Fig 1), from an initial rate of 97±5 and 94±9 breaths per minute respectively within the first ten minutes after induction to 84±6 and 78±6, respectively after 30 minutes. By 90 minutes, both groups had dropped to 75 breaths per minute (±5 under isoflurane and ±3 under alpha chloralose). There were no differences between treatment groups in breathing rate at any time point (F_(1,386)_ = 0.001, p = 0.97, Fig 1).

### 3.2 Heart rate decreases as anesthesia duration increases, with the greatest drop under alpha chloralose sedation

**Fig 2:**
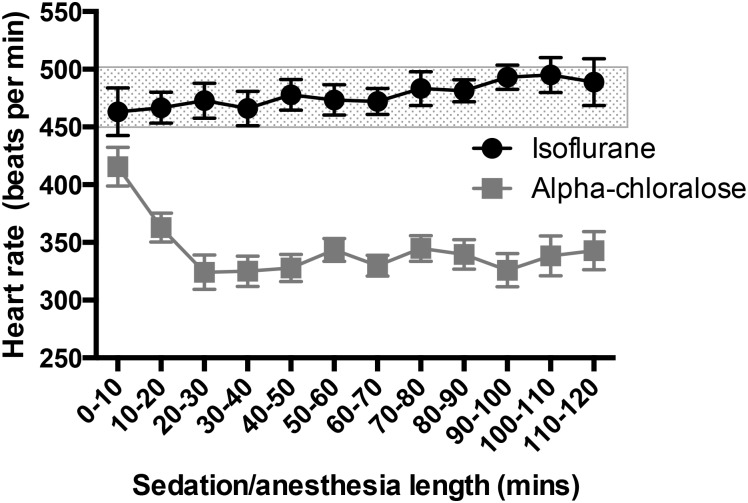
Heart Rate drops under alpha chloralose sedation. Mice retain a steady heart rate under isoflurane anesthesia, ranging from 463±20 beats/min to 489±20 beats/min. However, under alpha chloralose sedation, heart rate rapidly drops to below 350 beats/min, leading to a significant difference in heart rate between the treatment groups (F_(1,384)_ = 516.2,p<0.0001, n = 9–23 per time point). The shaded region indicates the normal heart rate for an awake mouse at rest (450–500 beats/min).

Normal heart rate for an awake mouse at rest is 450–500 beats/min, however there is great variability in the literature [15]. Under isoflurane anesthesia, heart rates remained above 450 beats/min, starting at 463±20 beats/min (0–10 minutes, Fig 2) and ending at 489±20 beats/min after two hours of anesthesia, and rates were stable throughout with little fluctuation (Time F_(11,384)_ = 1.26, p = 0.25). In contrast, initial heart rates were lower under alpha chloralose sedation, beginning at 415±17 beats/min immediately as sedation began, and dropping quickly to 320–350 beats/min after 20 minutes of sedation, as isoflurane was stopped and alpha chloralose absorbed. There was a significant difference between treatment groups (F_(1,384)_ = 516.2,p<0.0001, Fig 2).

### 3.3 Blood oxygenation levels do not differ between isoflurane anesthesia and alpha chloralose sedation

While the methods used to collect arterial blood may have minute levels of contamination from venous blood from local capillaries during blood collection from the tail artery, anecdotal evidence (data not published) from venous samples at experimental endpoints in a few mice confirmed expected partial pressures of oxygen in the range of P_a_O_2_<40mmHg. All partial pressures of oxygen analyzed as ‘arterial’ were >100 mmHg, confirming arterial blood collection.

**Fig 3:**
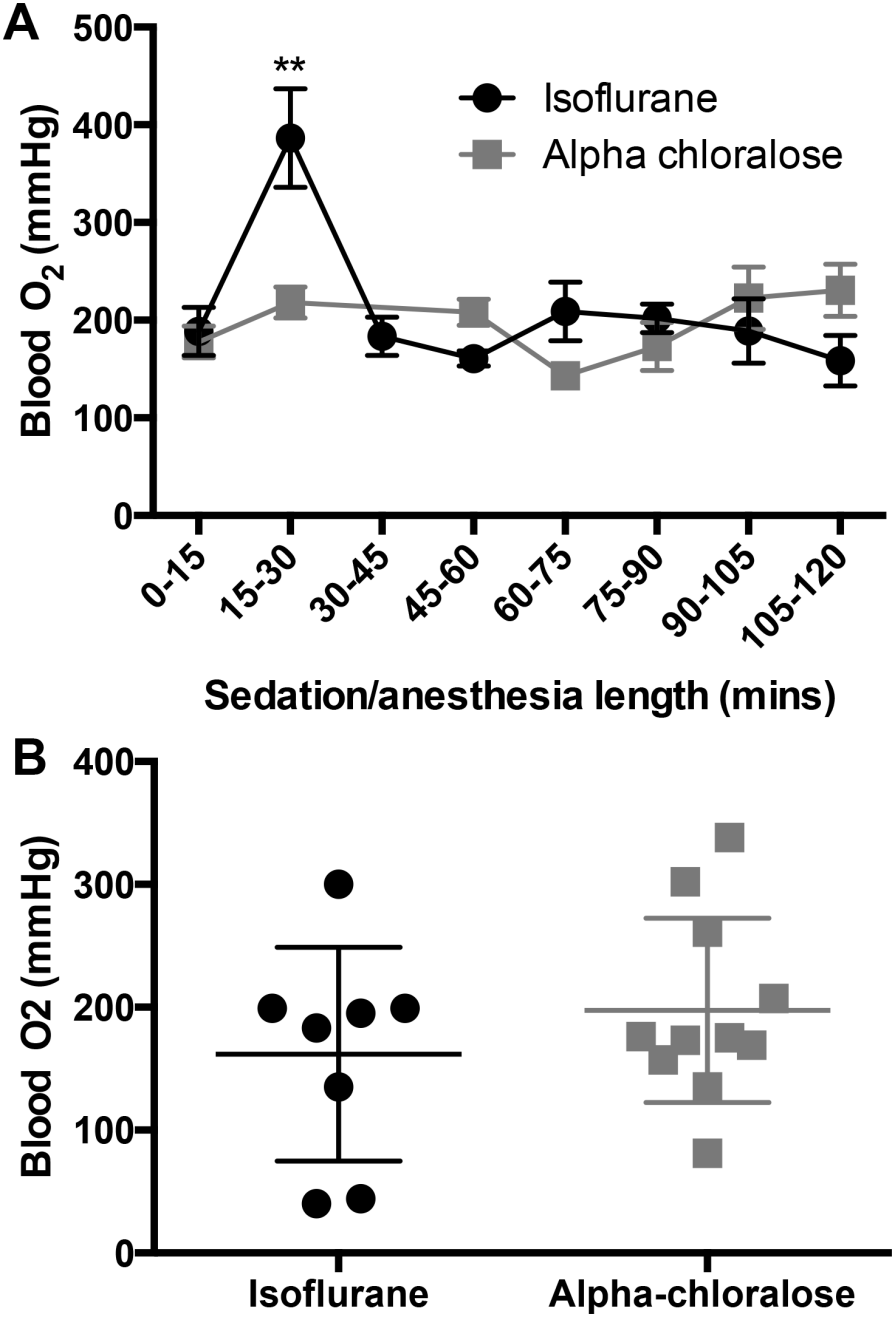
Blood oxygenation levels do not differ between isoflurane and alpha chloralose. A) Over the course of 120 minutes of sedation/anesthesia, there were no group differences in blood oxygenation levels (F_(1,81)_ = 0.6, p = 0.44, n = 4–19 per time point), but there was a significant effect of time in the isoflurane group, where blood oxygenation levels spiked between 15 and 30 minutes (F_(5,82)_ = 4.06, p = 0.002), then returned to baseline levels. B) Blood oxygenation is not different between isoflurane and alpha chloralose after 120 minutes of anesthesia/sedation (p = 0.67).

Arterial blood O_2_ levels were not different between treatment groups over the course of 120 minutes (F_(1,81)_ = 0.6, p = 0.44, Fig 3), but O_2_ levels spiked in the isoflurane-treated animals in the 15–30 minute time bin (p = 0.002) before returning to baseline levels (Fig 3a). After 120 minutes, O_2_ levels between groups were not different (p = 0.67, Fig 3b).

### 3.4 Carbon dioxide levels in arterial blood rise significantly under alpha chloralose sedation

**Fig 4:**
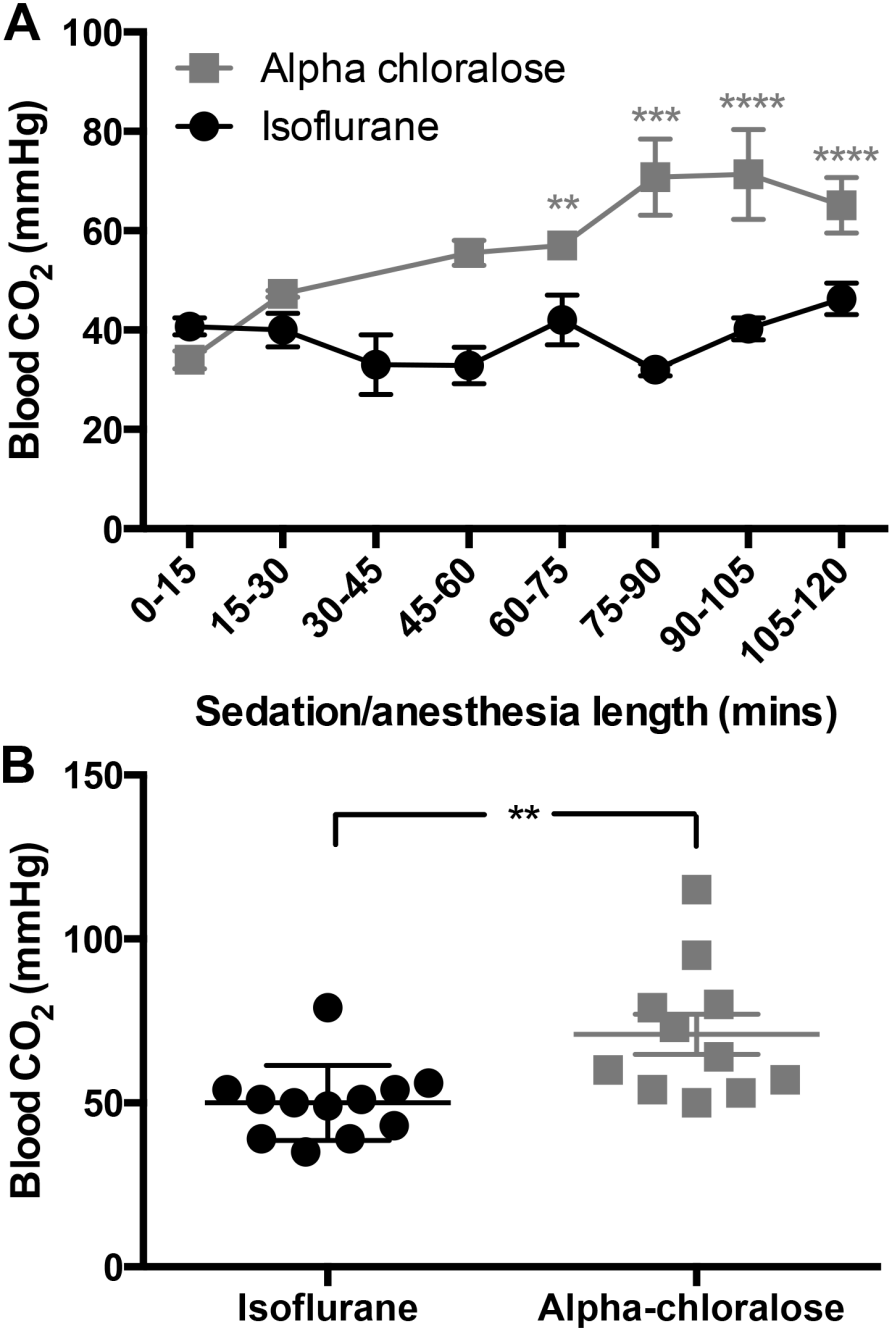
Blood carbon dioxide levels increase significantly under alpha chloralose sedation over the course of 120 minutes. A) Carbon dioxide levels in arterial blood were significantly higher under alpha chloralose sedation (F_(1,82)_ = 38.06, p<0.0001, n = 4–19 per time point). Although there was an overall effect of time (F_(5,82)_ = 4.06, p = 0.002), reflecting increasing CO_2_ levels over time, these levels did not differ significantly from baseline over the course of 120 minutes under isoflurane anesthesia (p>0.05). B) After 120 minutes, carbon dioxide levels are significantly higher in alpha chloralose-sedated animals (p = 0.002).

Over time, there is a significant overall increase in blood CO_2_ levels under both treatments (F_(5,82)_ = 4.06, p = 0.002, Fig 4), but this increase is significantly greater under alpha chloralose sedation (F_(1,82)_ = 38.06, p<0.0001, Fig 4a). After 120 minutes of sedation, arterial blood carbon dioxide levels were significantly higher in alpha chloralose-treated mice than isoflurane-treated mice (p = 0.002, Fig 4b).

### 3.5 Alpha chloralose sedation causes blood acidosis over time

**Fig 5:**
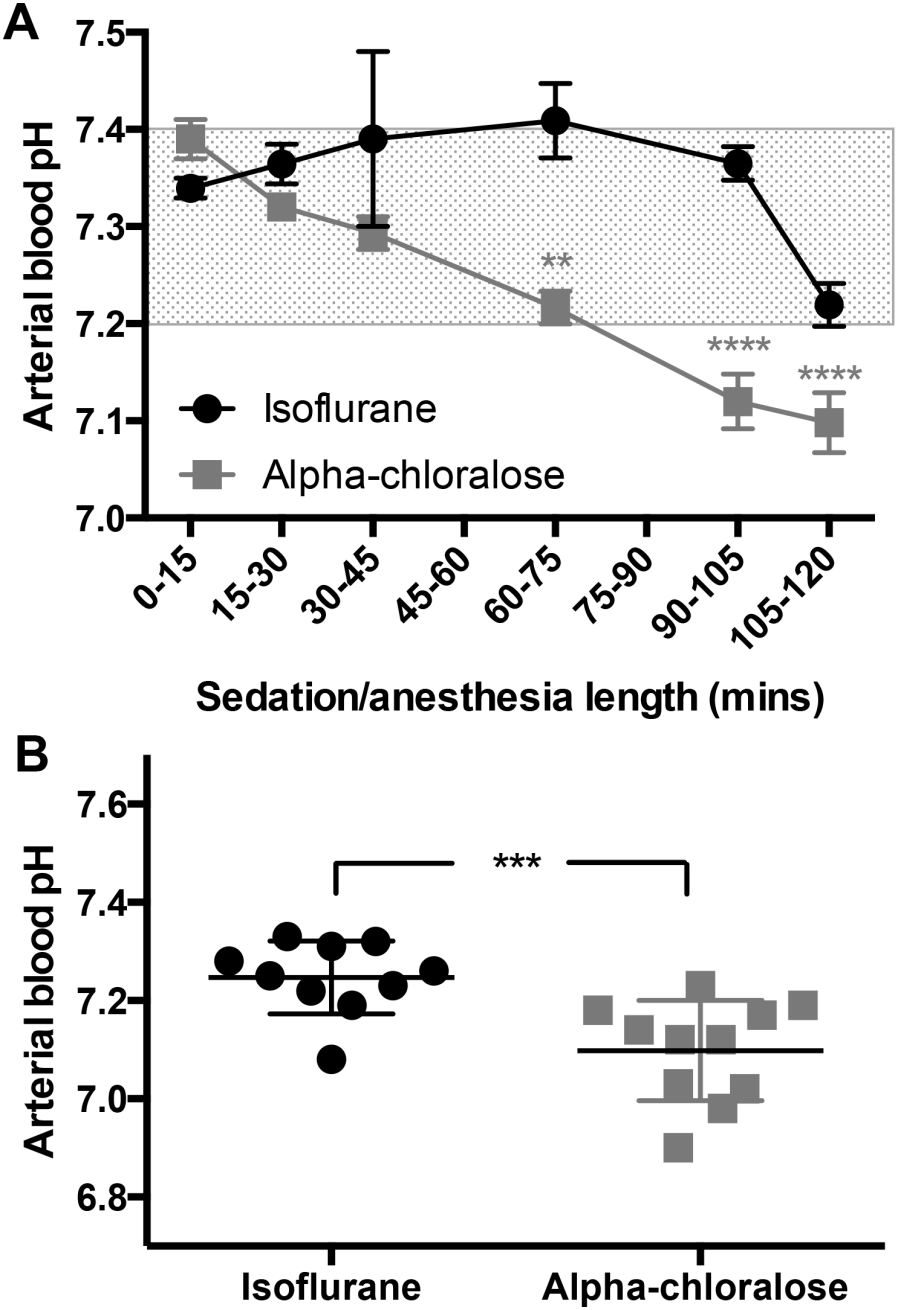
Alpha chloralose sedation causes clear blood acidosis. A) While pH drops under both types of anesthesia (F_(5,86)_ = 8.97, p<0.0001, n = 3–19 per time point), the drop is much more pronounced under alpha chloralose sedation (F_(1,86)_ = 35.72, p<0.0001), dropping from a normal reading of pH 7.39±0.05 during the first 15 minutes, to 7.13±0.07 by 120 minutes. In contrast, blood pH under isoflurane drops from 7.34±0.04 to 7.27±0.06 after two hours, which is still within a normal physiological range for the mouse. The shaded region indicates non-acidotic blood pH for the mouse (15). B) Blood is acidotic after 120 minutes of alpha chloralose sedation (p = 0.0006).

During the course of two hours of anesthesia/sedation, blood pH decreases significantly in both groups (F_(5,86)_ = 8.97, p<0.0001, Fig 5a), coinciding with an increase in blood CO_2_ levels. Despite the decrease, pH remains within a narrow and normal physiological range under isoflurane (pH 7.2–7.4)[15]. In contrast, alpha chloralose causes a significant drop in arterial blood pH within the first hour, and it continues to fall over time, showing a clear difference between treatment groups (F_(1,86)_ = 35.72, p<0.0001, Fig 5a).

At the 120 minute time point, blood is significantly more acidotic in mice under alpha chloralose sedation (p = 0.0006, Fig 5b). Under isoflurane, 9 of 10 mice still had blood pH levels within a normal range, but only 1 of 11 did not display acidosis (blood pH <7.2) under alpha chloralose.

## 4. Discussion

We have shown that isoflurane and alpha chloralose, both commonly utilized for brain imaging in mice [39, 40], cause differential effects on bodily homeostasis in the mouse. Alpha chloralose causes unstable physiological effects that could confound the outcome measures upon which functional brain imaging relies. Isoflurane anesthesia maintained a stable heart rate, blood pH and carbon dioxide partial pressure (P_a_CO_2_) as previously reported [41–43] despite an overall drop in breathing rate over 120 minutes. In contrast, alpha chloralose sedation caused a significant drop in breathing rates, a much-decreased but stable heart rate, and an increase in blood carbon dioxide levels (P_a_CO_2_) with a severe drop in arterial pH over time. Mice from both groups had no difference in blood oxygenation (P_a_O_2_).

Anesthesia is known to affect major organs, especially the cardiopulmonary systems. Anesthetized animals typically do not breathe as deeply as awake animals. Decreased tidal volumes coupled with decreased respiratory rates can result in hypoventilation and a build up of circulating CO_2_. Thus, respiratory acidosis (low pH and high P_a_CO_2)_ is not uncommon in anesthetized animals. Rao and Verkman [44] suggest a normal mouse partial CO_2_ pressure (P_a_CO_2_) of 34–35 mmHg. As CO_2_ levels increase under normal circumstances, central chemoreceptors sensitive to arterial CO_2_ tension cause a compensatory increase in respiration and the excess CO_2_ is exhaled to maintain normal blood gasses and pH [45]. Various drugs (including anesthetics) suppress the neurorespiratory center’s response to hypercapnia and are a common cause of respiratory acidosis. We found an increase in P_a_CO_2_ in both groups over time, but while the CO_2_ increase in isoflurane mice did not differ significantly from baseline values, the mice sedated with alpha chloralose showed a steady and significant rise in P_a_CO_2_. We suggest that the hypercapnia is most likely the result of the decreased respiratory rate exacerbating the anesthetic hypoventilation. These effects upon respiration could be prevented by artificially ventilating the animal, although the suitability of this invasive procedure will depend upon the experimental outcome measures under investigation.

While the mouse appears to have a wide range of normal pH in the literature [15, 41–44], acidosis in a human is diagnosed when pH falls below 7.36 [41]. Our data show that mice under isoflurane maintained a normal arterial pH for up to 2 hours under anesthesia. In contrast, the alpha chloralose mice were acidotic after only 30 minutes and pH steadily declined over time. The drop in pH was inversely related to a rise in P_a_CO_2_. We diagnose an acute primary respiratory acidosis by the low pH and high PaCO_2_. However, we cannot rule out a modest metabolic component to the degree of acidosis seen, as other metabolic changes secondary to anesthesia (lactate, insulin, glucose, free fatty acids, interleukin-6 and TNF-α levels) were not measured and others have seen elevated lactate in the anesthetized mouse [41, 46, 47].

The severe respiratory acidosis of alpha chloralose mice, demonstrated by the marked drop in pH, approached life-threatening levels. When pH drops below 7.2, metabolic enzymes may not function, hemoglobin’s binding affinity for oxygen is decreased, and cardiac arrhythmias may occur due to decreased cardiac contractility [19, 48]. These life-threatening conditions could have severe effects on CBF and BOLD outcome measures, thus interfering with functional imaging. Although the respiratory rate also decreased with isoflurane over time similar to alpha chloralose, we did not witness the same severity of P_a_CO_2_ elevation and the end drop in pH still remained within acceptable mouse limits.

The partial pressure of oxygen was high in both groups. Constantinides et al [43] found that the ideal level of inspired oxygen for maintenance of stable cardiovascular physiology in the mouse was above 50%. When breathing room air (~20% O_2_) the expected P_a_O_2_ is 95–100 mmHg with hemoglobin 95–98% saturated. With supplemental oxygen of 60% as in our study, the expected P_a_O_2_ is 250–300 mmHg [14] as shown in our results.

One major drawback with anesthetized rodents for functional brain scanning is that anesthesia significantly decreases cortical activation levels [33], which is the outcome that CBF and BOLD indirectly measures. However, lower levels of isoflurane anesthesia (0.75–1.5%) have proven to produce reliable cortical activation in the mouse [20, 22, 49]. Due to its limited effects on physiological measures, we therefore suggest that isoflurane is a more appropriate tool than alpha chloralose for mouse brain functional scanning.

Alpha chloralose, along with the muscle paralytic pancuronium bromide, has been utilized as an acceptable anesthetic for rat and mouse brain imaging [7, 27, 28, 40]. In both rats and mice, alpha chloralose cannot be reversed after administration, therefore correct initial dosages are critical, but still not guaranteed to produce similar results in all animals. Alpha chloralose has effects associated with respiratory complications, seizures and prolonged recovery in mammals (including dogs, swine and rodents)[32], and so it is not permitted by most animal care and use committees to be used for recovery experiments [50, 51].

While isoflurane is a known vasodilator [33], it has been recommended as a valuable alternative to alpha chloralose [52, 53]. We have also shown that it is capable of keeping a mouse physiologically stable over long periods of time. In particular, we show here that heart rate, P_a_O_2_, P_a_CO_2_ and blood pH is well maintained under isoflurane. This is a good indication of a well-functioning cardiopulmonary system and normal basal physiology.

The purpose of anesthesia is to provide restraint with minimal stress, pain or adverse side effects, and cause amnesia of painful procedures. Adequate agents work because they interfere with the brain’s conscious recognition of pain or stress. Therein lies the challenge of functional imaging of animals, because distress must be prevented while maintaining neural pathways of interest. Additionally, anesthetics cause secondary effects on other organs to various degrees, namely the cardiopulmonary systems. Alternative anesthetic and sedative agents have been successfully used for rodent functional imaging and include halothane [54], ketamine [55] and medetomidine [25, 56], although isoflurane and alpha chloralose remain the most utilized in the literature. However, all anesthetics contain caveats for brain imaging; for example, Schroeter et al [57] compared isoflurane, medetomidine sedation, propofol and urethane on BOLD and cerebral blood volume (CBV) imaging and saw that in the mouse, fMRI responses are influenced by stimulus-induced cardiac changes (an arousal response), potentially masking stimulus-evoked signals. Researchers must therefore carefully consider anesthetic choices based on the research question. We have shown that isoflurane does not interfere with some physiological measures that influence BOLD and CBF outcome measures. As it maintains physiological stability up to two hours, we suggest it is more suitable than alpha chloralose for functional imaging studies.

## Supporting Information

S1 Data All raw data used in this manuscript is contained within the Supporting Information file. (XLSX)
